# Acknowledgement to Reviewers of *Vaccines* in 2018

**DOI:** 10.3390/vaccines7010006

**Published:** 2019-01-08

**Authors:** Vaccines Editorial Office

**Affiliations:** MDPI, St. Alban-Anlage 66, 4052 Basel, Switzerland

Rigorous peer-review is the corner-stone of high-quality academic publishing. The editorial team greatly appreciates the reviewers who contributed their knowledge and expertise to the journal’s editorial process over the past 12 months. In 2018, a total of 80 papers were published in the journal, with a median time to first decision of 15 days and a median time to publication of 39.5 days. The editors would like to express their sincere gratitude to the following reviewers for their cooperation and dedication in 2018:

Admon, ArieLi, YizeAlexander-Miller, Martha A.Liang, BoAllain, Jean-PierreMangsbo, Sara M.Alugupalli, KishoreManini, IlariaAlvisi, GualtieroMartín-Acebes, MiguelAmarasinghe, GayaMemoli, Matthew J.Anderson, Larry JMiller, MattBarnier-Quer, ChristopheMoncunill, GemmaBaxter, AmyMurphy Schafer, AshleighBechini, AngelaNachbagauer, RaffaelBedford, HelenNakayama, TetsuoBerezin, EitanNiyonsaba, FrançoisBond, KatherineOomens, Antonius G. P. (Tom)Bowden, ScottParrish-Sprowl, JohnBrauner, AnneliePaterson, YvonneBrien, James D.Patton, JohnBrooke, Christopher ByronPetrini, StefanoBrown, Richard J. P.Pittau, MarcoBurns, JamesPizzo, ElioChevalier, ChristophePollard, AndrewChoi, YoukyungProw, NatalieChung, AmyProw, TralCobey, SarahRengarajan, JyothiCooper, ChristopherRogerson, StephenCorleis, BjörnRossi, ShannanCranfield, CharlesRossman, JeremyDaly, JanetRouiller, IsabelleDaniels, Calvin “Clay”Rouphael, Nadinede la Torre, Juan CarlosSaelens, XavierDe Vries, RorySaelens, XavierDelhon, GustavoSaijo, MasayukiEggink, DirkSavelyeva, NataliaEsser, MarkSchafer, Katherine MontagFerreira, FernandoSchotsaert, Michael A.Franzoni, GiuliaSheridan, BrianFuchs, WalterSikora, Aleksandra E.Geary, SeanSmith, AlysonGilbert, SarahStephens, EdwardGori, AlessandroStobart, ChristopherGowda, D. ChanneSutton, TroyGrodeland, GunnveigTam, Clarence CGrunwald, ThomasTan, Yee-jooHarfoot, RhodriThomas, Stephen JHarris, Audray K.Torner, NúriaHolst, JohanUlbert, SebastianHuang, XuefeiValkenburg, Sophie A.Huckriede, AnkeVanpouille-Box, ClaireIshikawa, TomohiroVeit, MichaelJackson, AndrewVesikari, TimoJassoy, ChristianWall, Katherine A.Kaljee, LindaWard, BrianKhaperskyy, Denys A.Wetzler, Lee M.Khatri, MaheshWikel, StephenKim, Shin-HeeWiley, Clayton A.Klein, MatthiasXie, HangKrauss, Isaac J.Xin, HongLafontaine, EricYamashita, MakotoLauring, AdamYeo, SeonjuLeclerc, DenisYuste, JoseLeitao, AlexandreZell, RolandLi, XuguangZhou, Fan

